# Whole-Genome Sequencing of 10 *Pseudomonas syringae* Strains Representing Different Host Range Spectra

**DOI:** 10.1128/genomeA.00379-15

**Published:** 2015-04-30

**Authors:** Claudia Bartoli, Sébastien Carrere, Jay Ram Lamichhane, Leonardo Varvaro, Cindy E. Morris

**Affiliations:** a Department of Science and Technology for Agriculture, Forestry, Nature and Energy (DAFNE), Tuscia University, Viterbo, Italy; b INRA, UR0407, Pathologie Végétale, Montfavet, France; c INRA, Laboratoire des Interactions Plantes-Microorganismes (LIPM), UMR441, Castanet-Tolosan, France; d CNRS, Laboratoire des Interactions Plantes-Microorganismes (LIPM), UMR2594, Castanet-Tolosan, France; e INRA, UAR 1240 Eco-Innov, BP 01, Thiverval-Grignon, France

## Abstract

*Pseudomonas syringae* is a ubiquitous bacterium that readily persists in environmental habitats as a saprophyte and also is responsible for numerous diseases of crops. Here, we report the whole-genome sequences of 10 strains isolated from both woody and herbaceous plants that will contribute to the elucidation of the determinants of their host ranges.

## GENOME ANNOUNCEMENT

The *Pseudomonas syringae* complex is composed of 13 phylogenetic groups (1) collectively able to cause disease on more than one hundred plant species (2, 3). *P. syringae* strains have also been isolated from substrates other than plants in various habitats linked to the water cycle. Many of these environmental strains, when tested in the laboratory, are also pathogenic for several plant species (4, 5). Here, we sequenced, assembled, and annotated the whole genomes of 10 strains of *P. syringae* isolated from diverse plants and representing 3 phylogenetic groups. These strains were chosen to represent different host range spectra based on the results of research to be reported elsewhere. From phylogroup 1, the strains included CFBP 1657 (pv. maculicola) isolated from *Brassica oleracea*, CFBP 1702 (pv. viburni) isolated from *Viburnum* sp., and PaVt10 (5) (pv. avellanae) isolated from *Corylus avellana*. From phylogroup 2, the strains were 41a from *Prunus armeniaca* and CFBP 1754 (pv. papulans) from *Malus sylvestris*. From phylogroup 3, we sequenced the genomes of strains CFBP 3205 (pv. amygdali) from *Prunus dulcis*, CFBP 3225 (pv. meliae) from *Melia azedarach*, CFBP 3226 (pv. dendropanacis) from *Dendropanax trifidus*, CFBP 4219 (pv. daphniphylli) from *Daphniphyllum* sp., and PseNe107 (5) (pv. savastanoi) from *Olea europaea*. With the exception of strain 41a, all other strains were from reference collections and were described previously. For all strains, DNA was extracted by using the Qiagen Genomic-tip 100/G kit after growing strains overnight at 26°C in a liquid nutrient broth. Illumina libraries were constructed with the NEXTflex PCR-free DNA sequencing kit and NEXTflex PCR-free barcodes, and genomes were sequenced by using MiSeq M00185 (250-bp paired-end reads). The insert sizes for each genome are reported in Table 1. Overall, the average insert size was 389 bp. *De novo* assembly was performed by using a pipeline that consists of a combination of Velvet (6), SOAP*denovo*, and SOAPGapCloser (7). The structural annotation of the contigs was achieved as previously described (8). The features for each genome are reported in Table 1. Analysis of the 10 *P. syringae* genomes showed that all strains present a complete type III secretion system.

**TABLE 1:** Genome characteristics

Strain name	Phylogroup	Accession no.	Genome size (bp)	Insert size (bp)	No. of contigs	*N*_50_ (bp)	No. of protein-coding genes	G+C content (%)
CFBP 1657	1	JYHH00000000	891,415,000	374	138	122,411	5,209	58.43
CFBP 1702	1	JYHK00000000	944,775,000	397	265	98,077	5,595	58.60
PaVt10	1	JYHC00000000	899,519,500	392	455	30,706	4,807	58.82
41a	2	JYHJ00000000	930,312,000	372	24	665,729	5,126	59.11
CFBP 1754	2	JYHI00000000	735,876,000	394	182	132,813	2,378	58.97
CFBP 3205	3	JYHB00000000	697,222,500	404	343	35,334	4,934	58.29
CFBP 3225	3	JYHE00000000	733,793,000	390	360	34,681	4,344	58.40
CFBP 3226	3	JYHG00000000	545,040,500	370	247	73,921	4,988	58.11
CFBP 4219	3	JYHD00000000	614,308,500	403	377	47,492	5,100	58.12
PseNe107	3	JYHF00000000	853,186,500	396	247	109,046	5,303	58.02

### Nucleotide sequence accession numbers.

These whole-genome shotgun projects have been deposited at DDBJ/EMBL/GenBank under the accession numbers listed in Table 1.
