# Correction for Bartoli et al., Whole-Genome Sequencing of 10 *Pseudomonas syringae* Strains Representing Different Host Range Spectra

**DOI:** 10.1128/genomeA.00628-15

**Published:** 2015-06-04

**Authors:** Claudia Bartoli, Sébastien Carrere, Jay Ram Lamichhane, Leonardo Varvaro, Cindy E. Morris

**Affiliations:** a Department of Science and Technology for Agriculture, Forestry, Nature and Energy (DAFNE), Tuscia University, Viterbo, Italy; b INRA, UR0407, Pathologie Végétale, Montfavet, France; c INRA, Laboratoire des Interactions Plantes-Microorganismes (LIPM), UMR441, Castanet-Tolosan, France; d CNRS, Laboratoire des Interactions Plantes-Microorganismes (LIPM), UMR2594, Castanet-Tolosan, France; e INRA, UAR 1240 Eco-Innov, BP 01, Thiverval-Grignon, France

## AUTHOR CORRECTION

Volume 3, no. 2, e00379-15, 2015. Page 1: Table 1 should appear as shown below.

**TABLE 1:** Genome characteristics

Strain name	Phylogroup	Accession no.	Genome size (bp)	Insert size (bp)	No. of contigs	*N*_50_ (bp)	No. of protein-coding genes	G+C content (%)
CFBP 1657	1	JYHH00000000	6,058,456	374	138	122,411	5,286	58.43
CFBP 1702	1	JYHK00000000	6,492,303	397	265	98,077	5,912	58.60
PaVt10	1	JYHC00000000	5,901,785	392	455	30,814	5,192	58.82
41a	2	JYHJ00000000	5,983,849	372	24	665,729	5,131	59.11
CFBP 1754	2	JYHI00000000	6,112,417	394	182	132,813	5,599	58.97
CFBP 3205	3	JYHB00000000	5,719,264	404	343	35,334	5,220	58.29
CFBP 3225	3	JYHE00000000	5,143,641	390	360	34,681	4,692	58.40
CFBP 3226	3	JYHG00000000	5,739,983	370	247	73,921	5,235	58.11
CFBP 4219	3	JYHD00000000	6,049,239	403	377	47,492	5,561	58.12
PseNe107	3	JYHF00000000	6,071,865	396	247	109,046	5,605	58.02

